# Supplementary material for: Estimation of Flood Inundation Area Using Soil Moisture Active Passive Fractional Water Data with an LSTM Model
Source: Sensors (Basel). 2025 Apr 16;25(8):2503. doi: 10.3390/s25082503 (PMC12030926; doi:10.3390/s25082503)
Supplement: Supplementary file 1 [file sensors-25-02503-s001.zip › sensors-3532987-supplementary.pdf]

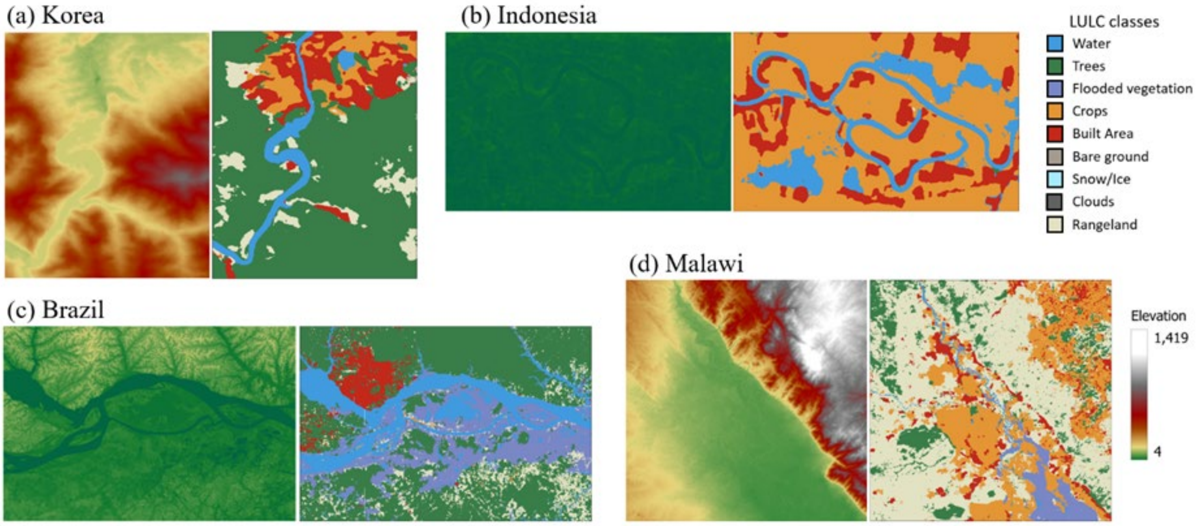

Figure S1. Elevation and LULC classes in the study area.

Table S1. The optimized hyperparameters

Hyperparameter	Search space	Description
Activation function	Tanh	Converts linear relationships into nonlinear ones
Hidden size	25	Modulates the output size of hidden layers in the LSTM network
Batch size	500	Number of samples processed by the neural network per iteration
Optimizer	Adagrad	Directs weight updates of the network
Learning rate	0.002	Controls the learning speed of the model

Table S2. Validation area extent

**Study area**	***w***	***h***	**Area extent**
Korea	180	216	36.72 to 36.78 N, 127.82 to 127.87 E
Indonesia	288	180	-7.01 to -6.96 S, 112.29 to 112.37 E
Brazil	3240	2160	-3.50 to -2.90 S, -60.20 to -59.30 W
Malawi	1800	1800	-16.40 to -15.90 S, 34.65 to 35.15 E
